# Mechanical Behavior of Polyurethane-Reinforced Coral Sand Under Unconfined Compression

**DOI:** 10.3390/polym18141744

**Published:** 2026-07-16

**Authors:** Linjian Ma, Hui Li, Fa Yang, Yanyan Cai, Hansheng Geng, Jian’an Wu

**Affiliations:** 1 State Key Laboratory of Disaster Prevention and Mitigation of Explosion and Impact, Army Engineering University of PLA, Nanjing 210007, China; patton.4400@163.com (L.M.); hsgeng@aeu.edu.cn (H.G.); uniquewujianan@163.com (J.W.); 2 Unit 96783 of PLA, Huaihua 418000, China; 3 College of Civil Engineering, Huaqiao University, Xiamen 361021, China; yycai@hqu.edu.cn

**Keywords:** coral sand, polyurethane, orthogonal experiment, microscopic mechanism

## Abstract

The low bearing capacity and high crushability of coral sand pose major challenges to island and reef foundation engineering. Herein, a newly developed non-isocyanate polyurethane was used to reinforce coral sand and the unconfined compressive behavior of polyurethane-reinforced coral sand was investigated based on an orthogonal experimental design. The effects of particle gradation, the mass ratio of polyurethane to sand and moisture content on strength, deformation, and energy evolution were analyzed. The results show that the highest uniaxial strength of 5.48 MPa was obtained for naturally graded coral sand with a polyurethane mass ratio of 30% under dry conditions. The moisture content was identified as the dominant factor affecting the strength, and elastic modulus of the reinforced samples. Increasing moisture content significantly reduced the crack initiation stress, dilation strength, peak strength and elastic modulus, while increasing Poisson’s ratio. In contrast, a higher polyurethane mass ratio improved the strength, stiffness and energy dissipation capacity, whereas particle gradation primarily influenced the crack initiation stress level. Under unconfined compression, the reinforced samples mainly exhibited ductile shear failure involving edge breakage, particle sliding, delamination and rupture of the cured polyurethane film. Microscopic observation indicated that the cured polyurethane worked as a surface film, an interparticle bridge and a pore-filling phase within the coral sand matrix. The enhancement in mechanical behavior was mainly associated with polymer bridging, pore filling, local interfacial adhesion and mechanical interlocking.

## 1. Introduction

With the continued expansion of marine resource development and island-reef engineering, increasing demands have been placed on foundation filling materials used in sea embankments, revetments, breakwaters, harbors and artificial islands. Coral sand, as a widely distributed in situ material in island and reef regions, has become an important material for shallow foundations and filling projects because of its local availability and low transportation cost [1,2]. However, island-reef engineering structures are continuously subjected to complex marine environmental actions, including waves, tides and typhoons, which may lead to degradation of foundation bearing capacity, local structural damage and deterioration of service performance. Therefore, in engineering scenarios such as post-disaster restoration, emergency reinforcement and rapid construction, there is an urgent need for foundation reinforcement materials capable of developing effective strength and bearing capacity within a short period.

Unlike conventional silica sand, coral sand is characterized by well-developed intraparticle pores and irregular particle morphology, leading to high porosity, strong compressibility and pronounced particle crushability [3,4,5,6,7,8]. These distinctive physical and mechanical characteristics result in low compactness and high brittleness of coral sand foundations, which may further cause insufficient bearing capacity, excessive post-construction settlement and reduced structural stability [9,10,11]. Therefore, rapid and efficient reinforcement of coral sand is of great significance for island-reef engineering, particularly where early strength development and rapid load-bearing performance are required.

In island and reef foundation engineering, inorganic binders are still commonly used for soil stabilization, among which cement has received the most attention [12,13,14,15,16,17]. Cement-based grouting has been examined in a number of studies on calcareous sand. For example, Fang et al. [18] carried out grouting simulation tests with cement slurry and reported improved reinforcement strength. The microstructural changes associated with cement treatment have also been discussed. Wang et al. [19] found that cement mortar modification can produce cementitious networks around coral sand particles, which contributes to volumetric stability. Similar observations were made by Zhang et al. [20], who showed that ultrafine cement grout can occupy interparticle voids and improve bonding between particles. Beyond single cement-based grouts, Zhang et al. [21] proposed a composite binder consisting of ultrafine Portland cement, nano-silica sol, fly ash and calcium sulfate whiskers; this system increased the compressive strength of cement-stabilized calcareous sand at different curing ages.

Although cement-based materials can improve the mechanical properties of coral sand, they still suffer from intrinsic limitations, including long setting time, slurry sedimentation, bleeding and drying shrinkage. These drawbacks are particularly unfavorable for emergency repair and rapid construction because delayed strength development prolongs construction waiting time and may induce uneven settlement or local instability before the foundation achieves sufficient bearing capacity. Therefore, polymer-based reinforcement materials, which can react at ambient temperature, cure rapidly and be applied conveniently, have emerged as promising alternatives to cement-based materials [22,23,24,25,26,27,28]. After penetrating the sand matrix, these materials bond adjacent particles and form three-dimensional polymer networks, thereby improving the strength and bearing capacity of the reinforced medium. Among the available polymer stabilizers, polyurethane has been widely discussed in geotechnical engineering because of its mechanical strength, low density, and expansion capability [29,30]. Existing efforts on polymer-stabilized sands have covered a broad range of polymer dosages. Polyurethane-treated calcareous sand and poorly graded sand have commonly adopted polyurethane contents of 5–20%, while much higher polyurethane proportions, up to 95%, have also been examined in polyurethane-foam-modified sand systems [31,32,33]. The curing regime is strongly dependent on the polymer system. Water-based polyurethane or organic-polymer-treated sand samples are commonly cured for 24–48 h before unconfined compression testing, while moisture-activated methylene diphenyl diisocyanate-based polymer systems may involve combined air–water curing for several days to promote strength development [34,35,36]. After curing, water-based polymer-treated sands generally show UCS values from several hundred kPa to about 1.2 MPa [34,35]. A higher polyurethane content can further improve the strength of treated sand. For example, in polyurethane-treated calcareous sand, the UCS increased from 0.203 to 1.836 MPa as the polyurethane content increased from 5% to 20% after approximately 24 h of curing [31]. However, the influence of water remains system-dependent. Depending on the polymer chemistry, water may facilitate polymer dispersion or moisture-triggered curing, whereas excessive water or post-curing immersion may soften polymer films, weaken interparticle bonding and reduce strength [36,37,38]. Mechanistically, polyurethane reinforcement restricts particle breakage and rearrangement by filling voids and cementing interparticle contacts, thereby improving the macroscopic strength and deformation resistance of the treated sand [39,40,41,42].

Although polyurethane-based stabilization has shown good potential for improving sand strength, most existing treated sand samples require curing periods of 24–48 h or even several days before sufficient strength can be achieved. Such curing durations may limit their applicability in emergency repair, rapid construction and island-reef engineering, where early strength development is critical. To address this limitation, in this study, a novel non-isocyanate polyurethane was developed to reinforce coral sand. Using an orthogonal experimental design, the effects of polyurethane content, moisture content and particle gradation on the mechanical behavior of the reinforced coral sand were systematically evaluated. Furthermore, scanning electron microscopy (SEM) analysis was employed to elucidate the microscale reinforcement mechanism of this newly developed material.

## 2. Materials and Experimental Procedure

### 2.1. Raw Materials and Mix Proportion

#### 2.1.1. New Polyurethane

The curing material used in this study was a two-component non-isocyanate polyure-thane-based binder, prepared by mixing Component A and Component B at a volume ratio of 1:1.15. Component A was a brown liquid with an initial viscosity of 9 mPa·s and a density of 0.925 g/mL. It was mainly composed of cyclic carbonate and epoxy resin at a ratio of 4:1 and served as the primary component responsible for the mechanical strength of the cured material. Component B was a colorless and transparent liquid with an initial viscosity of 6 mPa·s and a density of 1.075 g/mL. It mainly consisted of a compounded polyamine curing system, including diethylenetriamine, aromatic amine and long-chain aliphatic amine, which was used to adjust the system viscosity and promote the penetration of the binder into sandy soil pores. After Components A and B were mixed, the system maintained a relatively low viscosity, facilitating sufficient penetration of the binder into the pore space of sandy soil [43,44,45].

**Figure 1:**
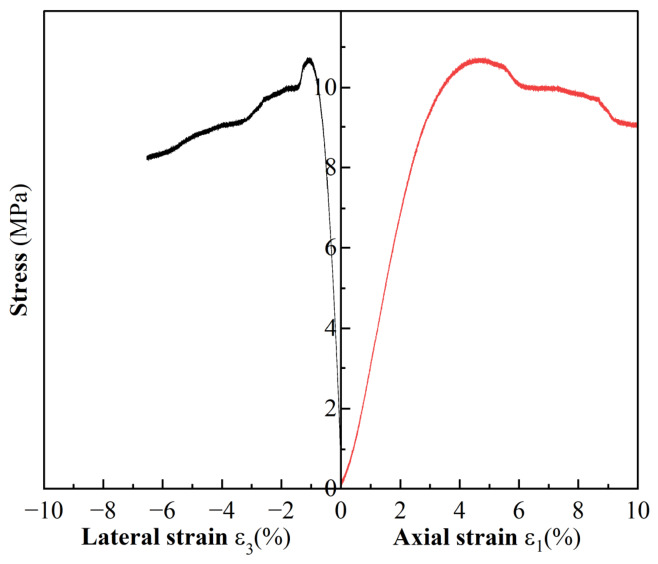
Stress–strain curves of pure polyurethane under unconfined compression.

As shown in Figure 1, the samples demolded after 90 min of curing reached a compressive strength of 10.7 MPa and an elastic modulus of 0.38 GPa, with a Poisson’s ratio of 0.25. This result indicates that the polymer developed strength within a relatively short curing period. The post-peak stress–strain response shows pronounced ductility, with a gradual stress decline accompanied by continuous strain accumulation. Based on these mechanical characteristics, the material can be regarded as a high-strength elastoplastic polymer.

#### 2.1.2. Coral Sand

**Figure 2:**
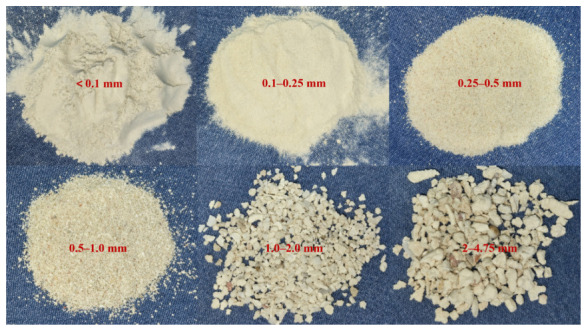
Raw coral sand with different size fractions.

The coral sand, sourced directly from the South China Sea islands, exhibits yellow coloration with intermingled macro-sized coral debris. The raw coral sand was sieved and fractionated into six particle size ranges: 0–0.1 mm, 0.1–0.25 mm, 0.25–0.5 mm, 0.5–1.0 mm, 1.0–2.0 mm and 2.0–4.75 mm, as illustrated in Figure 2.

**Figure 3:**
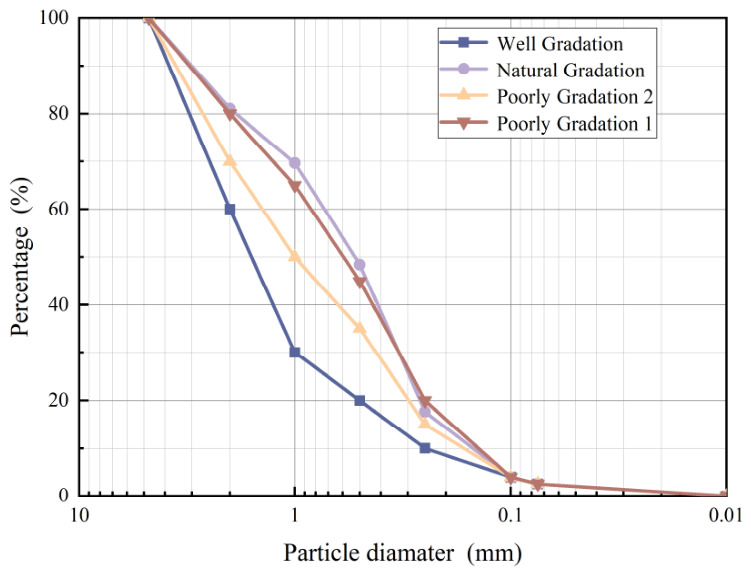
Particle size distribution of coral sand.

By mixing the sieved coral sand in different proportions, four types of graded coral sand were prepared, namely poorly graded sand 1, poorly graded sand 2, well-graded sand and naturally graded sand. The grain size distribution curves of each graded coral sand are shown in Figure 3. The basic parameters used to characterize particle gradation, including density, void ratio, coefficient of uniformity *C*_u_, coefficient of curvature *C*_c_ and *d*_50_, were measured or calculated according to the Chinese standard GB/T 50123-2019 [46]. The results are listed in Table 1. As stipulated in Chinese Standards GB/T 50145-2007 [47], when the parameters meet both *C*_u_ > 5 and 1 < *C*_c_ < 3 the coral sand can be classified as well-graded sand.

### 2.2. Sample Preparation

**Figure 4:**
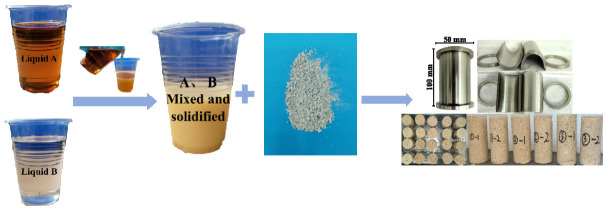
Process of sample production.

The constituent materials were homogenized using a mechanical mixer following a sequential addition protocol. A total of 250 g of coral sand substrate was first loaded, followed by the premixed two-component polyurethane formulation consisting of liquid Components A and B. After 60 s of controlled mixing, the composite was transferred into cylindrical molds (Φ50 mm × 100 mm). To ensure proper interfacial lubrication, the inner surfaces of the molds were coated with vaseline and lined with 0.1 mm polytetrafluoroethylene films, complemented by polytetrafluoroethylene base plates for enhanced demolding efficiency. Given the fragile nature of coral sand particles, the mold walls were gently tapped with a rubber mallet during casting to optimize mixture compaction. The cast surface was subsequently leveled using a geotechnical trimming knife to minimize stress concentration during testing. The confined molds were then cured under dimensional restraint for 2 h prior to demolding. Figure 4 schematically illustrates the sample fabrication process.

### 2.3. Experimental Procedure

**Figure 5:**
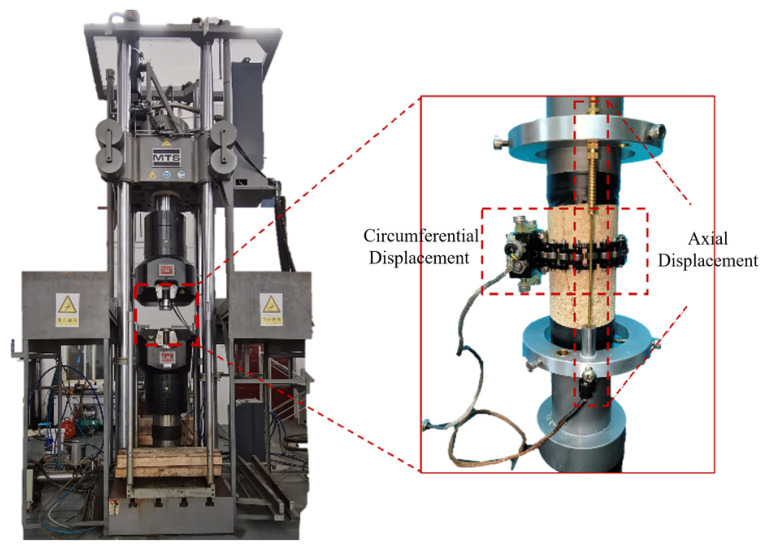
Test apparatus.

The MTS 647.250 electro-hydraulic servo-controlled testing system was used to conduct the unconfined compression tests. The system consists of a loading frame, a hydraulic power unit and a control system. The maximum static loading capacity was 2500 kN, with a load resolution of 10 N. Displacement-controlled loading was applied at a rate of 0.005 mm/s. As shown in Figure 5, the axial deformation of the sample was measured using a linear variable displacement transducer (LVDT), while the circumferential deformation was monitored using a mechanical chain sensor.

A three-factor four-level orthogonal experiment (Table 2) was designed to investigate the effects of the mass ratio of polyurethane to sand, the moisture content and particle gradation on consolidation performance. Experimental validation by trial and error determined that the minimum polyurethane mass ratio threshold for maintaining sample integrity post-demolding is 15%. As detailed in Table 3, the experimental matrix consists of 16 test groups with duplicate samples per group, resulting in 32 samples dedicated to unconfined compression testing. The 16 groups in the orthogonal experimental design are assigned identifiers in the format “gradation group-polyurethane mass ratio-moisture content.” For example, poorly graded sand 1, 25% polyurethane mass ratio and 10% moisture content are marked as P1-C25-W10.

## 3. Results and Analysis

### 3.1. Stress–Strain Curves

**Figure 6:**
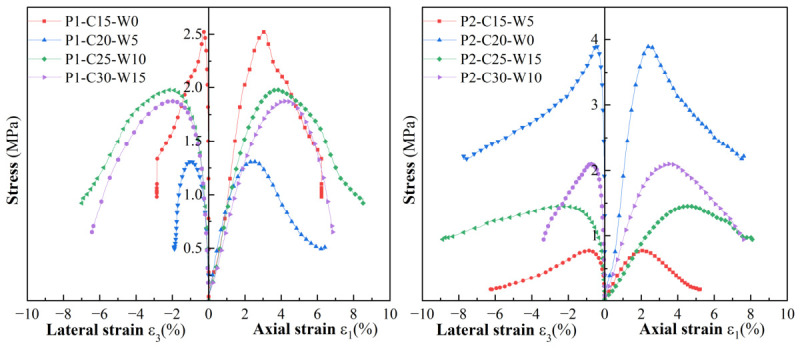

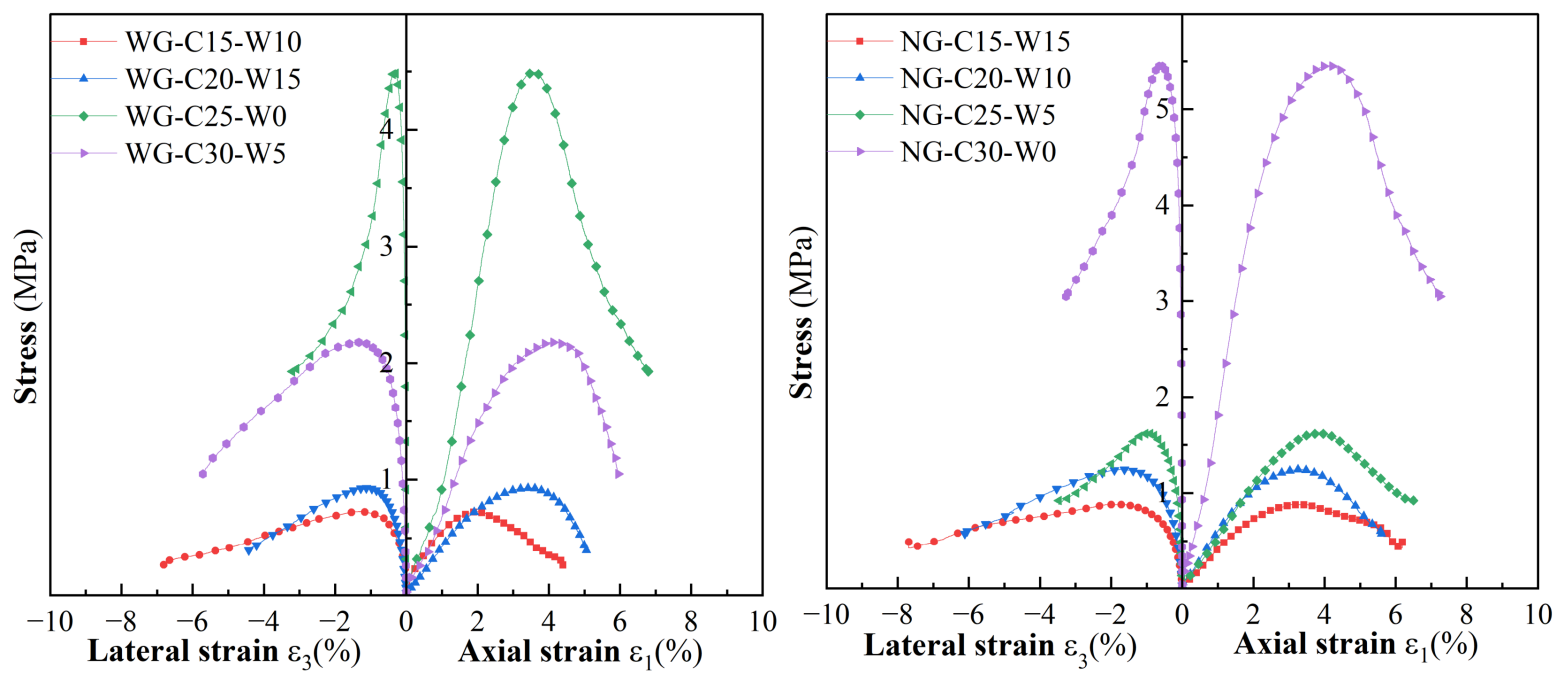
Stress–strain curves of polyurethane-reinforced coral sand samples under unconfined compression.

Figure 6 shows the stress–strain curves of polyurethane-reinforced coral sand samples under unconfined compression. All the stress–strain curves exhibit a similar shape, which can be divided into four distinct stages: the compaction stage, elastic deformation stage, crack propagation stage and the post-peak residual stage. In the compaction stage, the stress–strain curve shows a downward trend as internal pores gradually close and compact. During the elastic deformation stage, the curve becomes approximately linear with increasing load. At this stage, the sample deformation is primarily governed by mutual compression between particles, while the polymer-cured membrane constrains their relative movement. In a dry state, the polyurethane-reinforced coral sand sample exhibits obvious strength enhancement and smaller peak lateral strain. With increasing moisture content, the stress–strain curve becomes broader and flatter. As the applied load rises further, the sample enters the crack propagation stage. At this stage, particle displacement increases, interparticle interaction weakens and some particles undergo shearing or crushing. In the post-peak residual stage, the polyurethane network reaches its ductile limit and begins to rupture, followed by a rapid decline in stress and the development of macroscopic cracking or failure in the sample. Once the stress decreases to a certain level, the rate of decline becomes lower, indicating that the reinforced coral sand retains a certain degree of ductility under the rapid curing condition of the newly developed polyurethane binder.

Further analysis of the stress–strain curves allows for the quantitative extraction of fundamental mechanical parameters at different deformation stages. The calculated parameters include crack initiation stress, dilation strength, peak strength, elastic modulus, Poisson’s ratio, absorbed energy and dissipated energy. The results for each polyurethane-reinforced coral sand sample are summarized in Table 4.

### 3.2. Strength Property

#### 3.2.1. Crack Initiation Stress

The polyurethane-reinforced coral sand is a heterogeneous material, where micro-pores and particle boundaries induce local stress concentrations under loading. As the local stress exceeds the material strength, micro-failures occur, transferring stress to the crack tip and promoting further propagation.

The crack volume strain method is widely used to analyze rock and granular material failure [48]. The total volumetric strain *ε*_v_ is the sum of axial and lateral strains (*ε*_1_ + 2*ε*_3_) and can be divided into elastic strain $\varepsilon_{v}^{e}$ and crack-induced strain $\varepsilon_{v}^{c}$ caused by crack closure or propagation:

(1) $$\varepsilon_{v}=\varepsilon_{1}+2\varepsilon_{3}$$

(2) $$\varepsilon_{v}^{e}=\frac{1-2v}{E}(\sigma_{1}+2\sigma_{3})$$

(3) $$\varepsilon_{v}^{c}=\varepsilon_{v}-\frac{1-2v}{E}(\sigma_{1}+2\sigma_{3})$$

**Figure 7:**
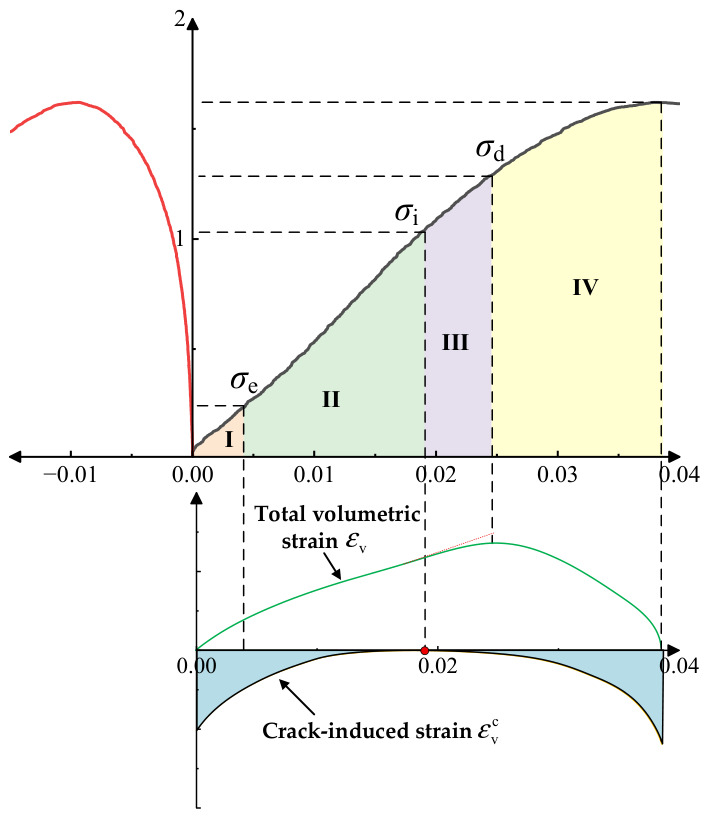
Determination of the crack initiation stress.

As shown in Figure 7, under zero confining pressure (*σ*_3_ = 0), the sample first undergoes crack closure and elastic compression with increasing axial strain, during which the total volumetric strain gradually increases, indicating overall compaction. With further loading, cracks initiate and propagate stably, and the total volumetric strain curve gradually deviates from the linear stage. Meanwhile, the crack volumetric strain curve departs from the nearly horizontal baseline and exhibits a distinct turning point, marked by the red dot in Figure 7. The axial strain corresponding to this point is projected onto the axial stress–strain curve, and the corresponding axial stress is defined as the crack initiation stress *σ*_i_. This stress represents the critical stress for the transition from the linear elastic stage to the stable crack propagation stage. As loading continues, the total volumetric strain reaches its maximum compaction, and the corresponding axial stress is defined as the dilatancy stress *σ*_d_. Therefore, *σ*_d_ characterizes the critical stress at which the sample deformation changes from compaction-dominated to dilation-dominated and also reflects the transition of crack evolution from stable propagation toward unstable growth. The ratio K defined as the ratio of crack initiation stress to peak strength, namely the crack initiation stress level, reflects the material heterogeneity, with smaller K values indicating greater non-uniformity.

(4) $$K=\frac{\sigma_{i}}{\sigma_{c}}$$

where *σ*_c_ is the peak strength. The parameter *K* reflects the relative stress level of crack initiation and the material heterogeneity. A smaller *K* indicates earlier crack initiation and stronger material heterogeneity.

The effects of gradation, the mass ratio and the moisture content on crack initiation stress and crack initiation stress level were evaluated using analysis of variance (ANOVA), as shown in Table 5. In the ANOVA table, SS denotes the sum of squares, which reflects the amount of variation attributed to each factor or to the experimental error. DOF denotes the degree of freedom associated with each source of variation. The *F*-value represents the relative importance of each factor compared with the experimental error and a larger *F*-value generally indicates a stronger factor effect. The *p*-value is used to determine whether the effect of a factor is statistically significant. The significance levels are denoted as follows: *ns* indicates no statistical significance; (*) indicates a significant effect (p<0.05); (**) indicates a highly significant effect (p<0.01); and (***) indicates an extremely significant effect (p<0.001).

For crack initiation stress, the moisture content showed the most significant effect, with an *F*-value of 24.51 and a *p*-value of 0.001. The mass ratio also had a statistically significant effect, with an *F*-value of 10.038 and a *p*-value of 0.009. In contrast, the effect of gradation was not statistically significant, with an *F*-value of 1.96 and a *p*-value of 0.221. These results indicate that the moisture content is the dominant factor controlling crack initiation stress, followed by the mass ratio, whereas the influence of gradation is relatively limited. For the crack initiation stress level, gradation showed the most significant effect, with an *F*-value of 95.25 and a *p*-value lower than 0.001. The effects of moisture content and mass ratio were also statistically significant, with *p*-values of 0.01 and 0.013, respectively. Therefore, unlike the crack initiation stress, the crack initiation stress level is more sensitive to particle gradation.

**Figure 8:**
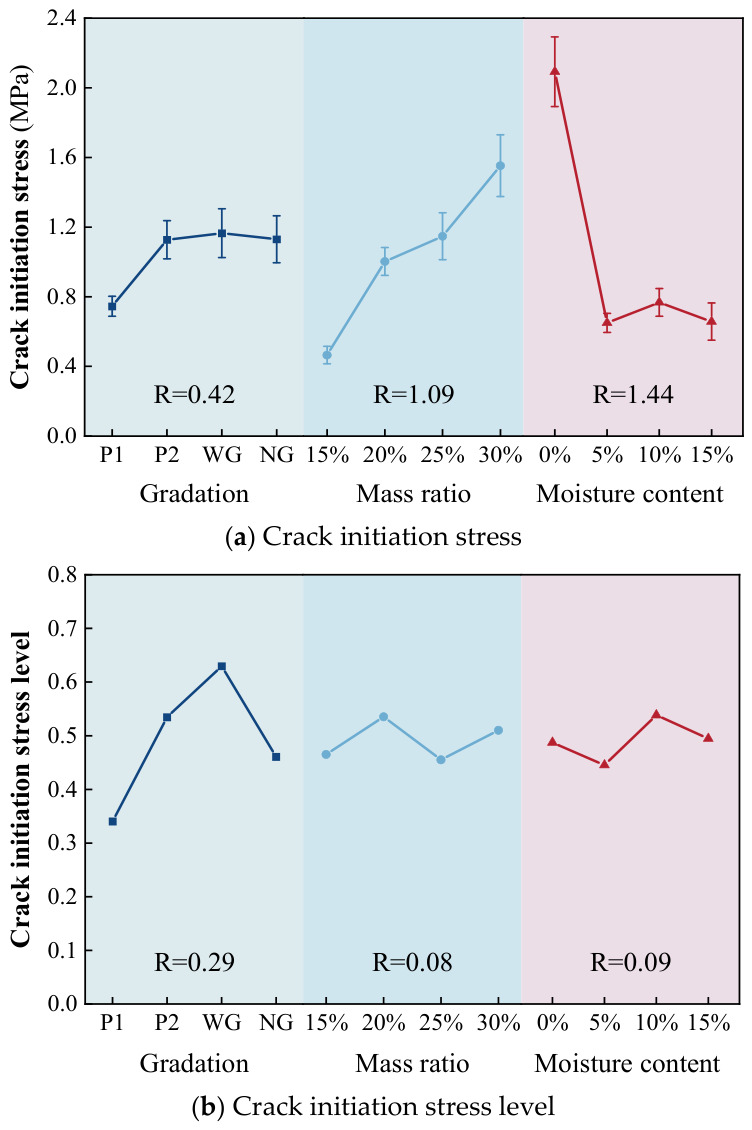
Sensitivity of crack initiation stress to various factors.

Figure 8 presents the variations in crack initiation stress under different influencing factors. For crack initiation stress, the calculated range values for gradation, the mass ratio and the moisture content are 0.42, 1.09 and 1.44, respectively. It is observed that the P1 gradation exhibits the lowest crack initiation stress, while the other gradations (NG, P2 and WG) show relatively close values. With an increasing the mass ratio, the crack initiation stress increases correspondingly, whereas higher moisture content results in a gradual reduction in crack initiation stress. For the crack initiation stress level, the calculated range values for gradation, the mass ratio and the moisture content are 0.29, 0.08 and 0.09, respectively, indicating that gradation is the most sensitive factor among the three. The crack initiation stress level differs among the gradation groups and follows the order P1 < NG < P2 < WG. This result may be associated with the well-graded particle distribution, which allows polyurethane to form a relatively uniform and compact internal structure during the reinforcement process.

This phenomenon can be attributed to the well-graded structure of the WG samples. In these samples, tighter interlocking between coarse and fine particles reduces porosity and provides a more stable particle framework. During polyurethane infiltration and curing, this framework is more favorable for the formation of a continuous cementation network, which helps distribute internal stress more evenly and delays crack initiation. Poorly graded samples, by comparison, contain more irregular pore structures and larger particle size disparities. During polymer curing, resin-rich and resin-deficient zones are more likely to form, leading to nonuniform bonding-film thickness and heterogeneous interfacial strength. This microstructural heterogeneity increases local stress concentration and promotes earlier crack initiation in polyurethane-reinforced coral sand under external loading.

#### 3.2.2. Dilation Strength

**Figure 9:**
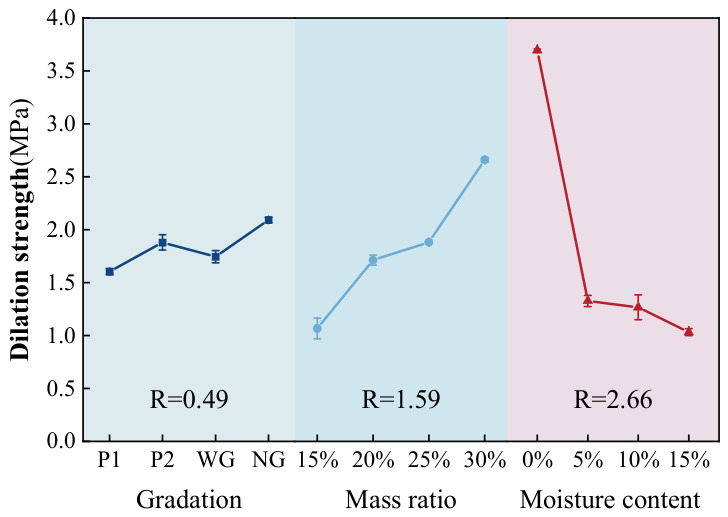
Sensitivity of dilation strength to various factors.

As shown in Table 6, the moisture content and the mass ratio had statistically significant effects on dilation strength, whereas the effect of gradation was not significant. Figure 9 further show that dilation strength increased with an increasing mass ratio but decreased markedly with increasing moisture content. When the mass ratio reached 30%, the dilation strength of the sample was 2.49 times higher than that of the sample with a 15% mass ratio, whereas an increase in the moisture content leads to a marked reduction in dilation strength. The dilation strength of the sample without moisture content was 3.58 times higher than that of the sample with 15% moisture content.

#### 3.2.3. Peak Strength

The ANOVA results for peak strength are presented in Table 7. The moisture content had the most significant effect on peak strength, with an *F*-value of 47.85 and a *p*-value lower than 0.001. The mass ratio also had a significant effect, with an *F*-value of 13.32 and a *p*-value of 0.0046. These results demonstrate that the peak strength is mainly governed by moisture content and the polyurethane-to-sand mass ratio.

**Figure 10:**
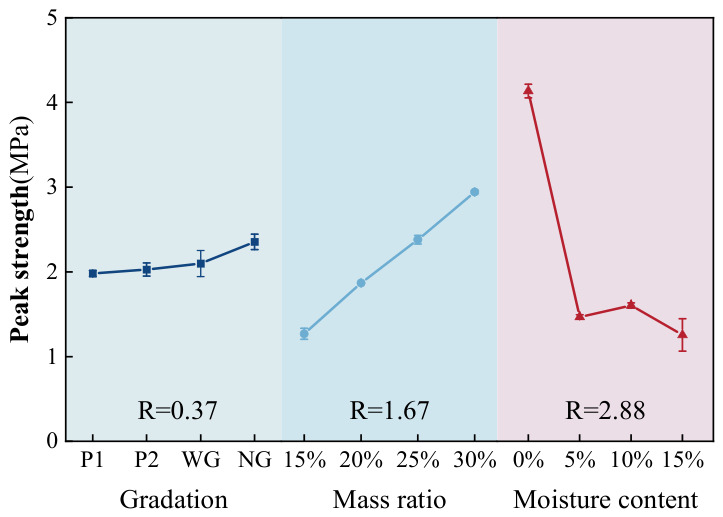
Sensitivity of peak strength to various factors.

Figure 10 illustrates the variation in peak strength influenced by different factors. The calculated range values for gradation, the mass ratio and the moisture content are 0.37, 1.67 and 2.88, respectively. Among these, the moisture content shows the largest range, indicating that it has the most significant effect on peak strength. As shown in Figure 10, the peak strength decreases with increasing moisture content, with the most pronounced reduction occurring during the transition from the dry to the low-moisture condition. In comparison, the peak strength increases with the polyurethane mass ratio, whereas changes in gradation produce only minor effects. The influence trends of mass ratio and moisture content on peak strength are consistent with their respective effects on crack initiation and dilation strength. This phenomenon can be attributed to the fact that a higher polyurethane dosage allows more effective filling of the interparticle pores within the polyurethane-reinforced coral sand and promotes the formation of a thicker and more continuous polymer membrane. Such a structure may enhance interparticle bonding, reduce internal defects and improve the load-bearing capacity of the sample. When the moisture content is too high, polymer curing becomes less complete and the interfacial adhesion between the polyurethane film and coral sand particles is weakened. As a result, the mechanical strength of the sample decreases.

### 3.3. Deformation Property

#### 3.3.1. Elastic Modulus

**Figure 11:**
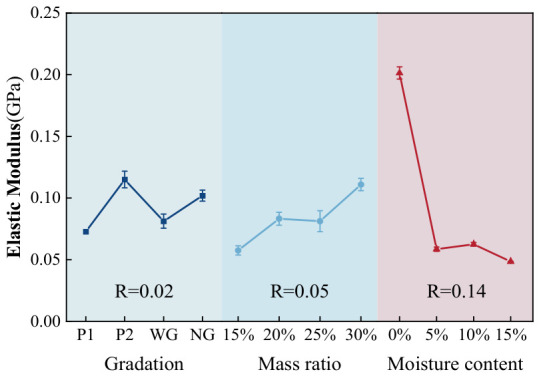
Sensitivity of elastic modulus to various factors.

As shown in Table 8, the moisture content had a statistically significant effect on the elastic modulus, whereas the effects of gradation and mass ratio were not significant. Figure 11 further reveal that the elastic modulus decreased markedly with increasing moisture content. The elastic modulus tended to increase with an increasing mass ratio. This increase may be related to the formation of a thicker and more continuous cured polyurethane film within the sample. The polymer film strengthens interparticle bonding and improves the deformation resistance of the reinforced coral sand. By contrast higher moisture content, may interfere with bonding between coral sand particles and the polyurethane matrix, weakens interfacial adhesion, and reduces the structural integrity of the composite.

#### 3.3.2. Poisson’s Ratio

**Figure 12:**
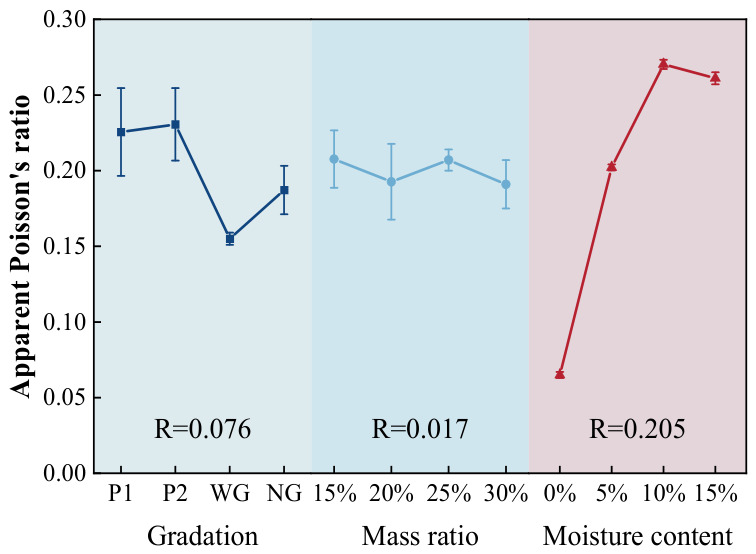
Sensitivity of apparent Poisson’s ratio to various factors.

As shown in Table 9, the moisture content had a statistically significant effect on the apparent Poisson’s ratio, whereas the effects of gradation and mass ratio were not significant. Figure 12 shows that the apparent Poisson’s ratio increased markedly with increasing moisture content. In particular, the apparent Poisson’s ratio at 15% moisture content was much higher than that under dry conditions. An increase in moisture content reduces the interfacial friction between coral sand particles and the surrounding polyurethane matrix. Under axial loading, this condition allows greater lateral deformation of the reinforced sample. The increase in apparent Poisson’s ratio with moisture content indicates that volumetric dilation of the sample is more readily mobilized at higher moisture content. Under radial confinement, the suppression of volumetric dilation may lead to a more pronounced enhancement in peak strength.

### 3.4. Energy Dissipation

The stress–strain curve was used to characterize the macroscopic deformation and energy evolution of the sample. The sample is assumed to deform without heat exchange with the surrounding environment. The external work input per unit volume is given by dU=σdε. Hence, the area under the loading branch of the stress–strain curve represents the absorbed strain energy density. The absorbed energy was partitioned into recoverable elastic strain energy and dissipated energy according to the energy balance, as follows:

(5) $$U=U_{E}+U_{D}$$

where *U* is the absorbed energy, *U*_E_ is the elastic strain energy and *U*_D_ is the dissipated energy.

**Figure 13:**
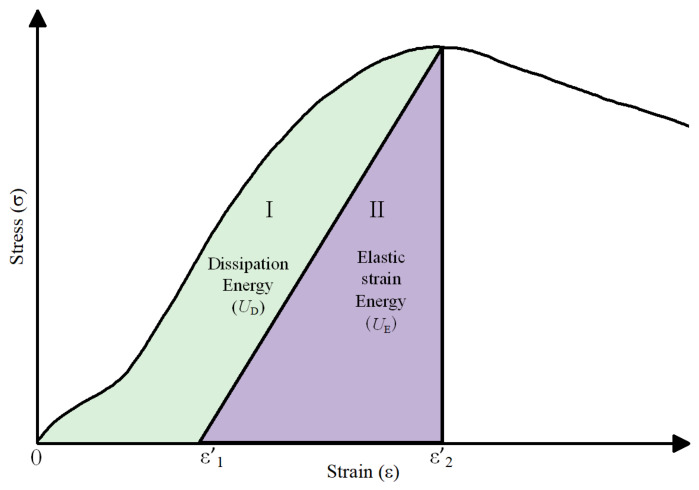
Schematic illustration of energy components in the stress–strain curve under unconfined compression.

As shown in Figure 13, area I, obtained from the triangular area bounded by the assumed linear elastic unloading path, the vertical line at $\varepsilon_{2}^{\prime }$ and the strain axis denotes the estimated dissipated energy *U*_D_. Area II denotes the estimated recoverable elastic strain energy *U*_E_, calculated as the difference between the absorbed strain energy *U* and *U*_E_:

(6) $$U_{D}=U-U_{E}=\int_{0}^{\varepsilon_{2}}\sigma_{1}d\varepsilon_{2}-\int_{\varepsilon_{1}^{\prime }}^{\varepsilon_{2}^{\prime }}\sigma_{1}d\varepsilon^{\prime }$$

The effects of the three factors on energy-related parameters were evaluated using ANOVA, as shown in Table 10. For dissipated energy, the mass ratio showed a statistically significant effect, with an *F*-value of 6.31 and a *p*-value of 0.028. For the energy dissipation ratio, none of the three factors showed a statistically significant effect. Gradation had an *F*-value of 0.59 and a *p*-value of 0.643, the mass ratio had an *F*-value of 0.65 and a *p*-value of 0.611, and the moisture content had an *F*-value of 1.53 and a *p*-value of 0.301. These results indicate that the energy dissipation ratio is not significantly affected by gradation, mass ratio, or moisture content.

**Figure 14:**
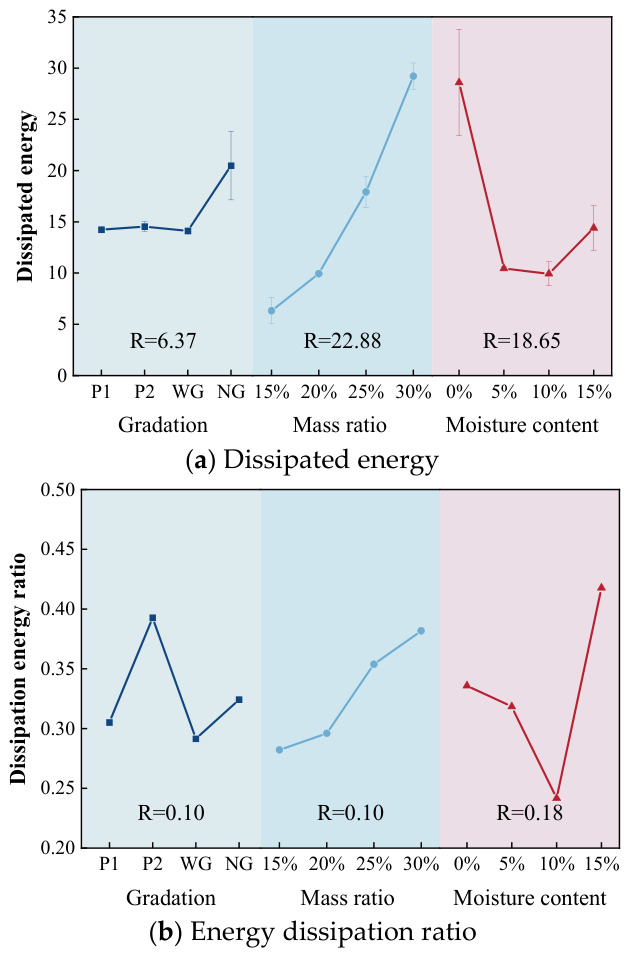
Sensitivity of energy dissipated to various factors.

Figure 14 further illustrates the effects of various factors on the energy characteristics of the reinforced samples. The results reveal that dissipated energy and the energy dissipation ratio increase with the rise in mass ratio. This observation indicates that a denser and more continuous polymer network formed at higher mass ratio enhances interparticle bonding and leads to a more uniform stress distribution. Consequently, the samples may sustain greater deformation before failure occurs. As the mass ratio increases, the absorbed energy also increases. Since the dissipated energy increases at a higher rate than the absorbed energy, the energy dissipation ratio also shows an increasing trend. One may further postulate that under cyclic loading, a higher mass ratio may lead to a denser polymer network and a more stable energy-dissipated path, thereby potentially delaying cumulative deformation and stiffness degradation during repeated loading.

### 3.5. Failure Pattern

**Figure 15:**
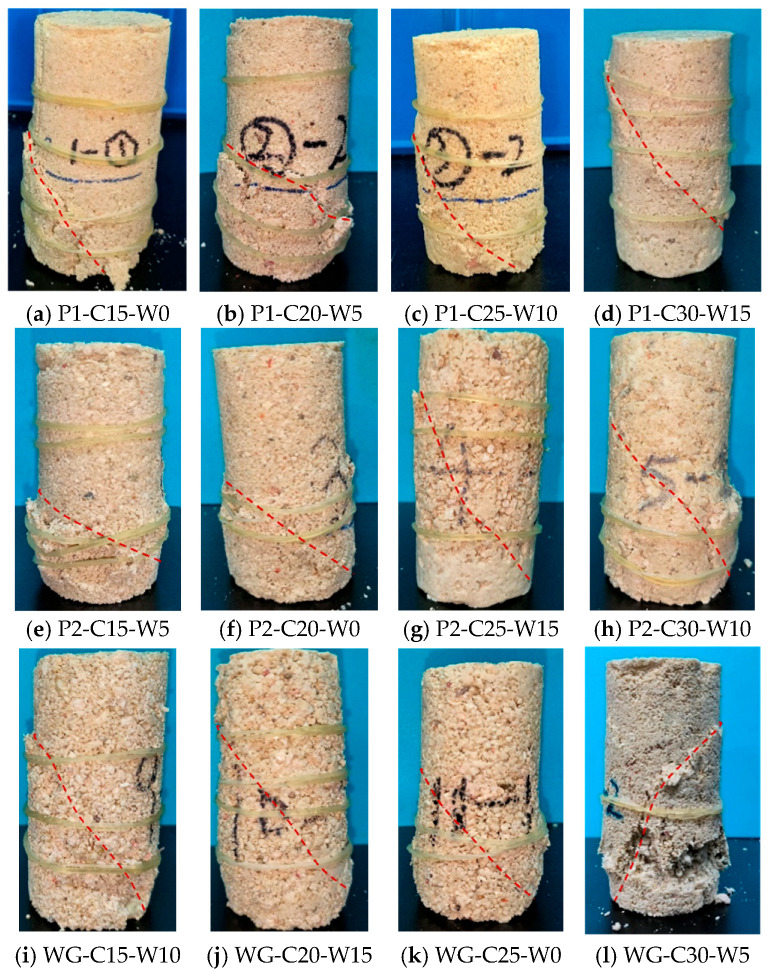

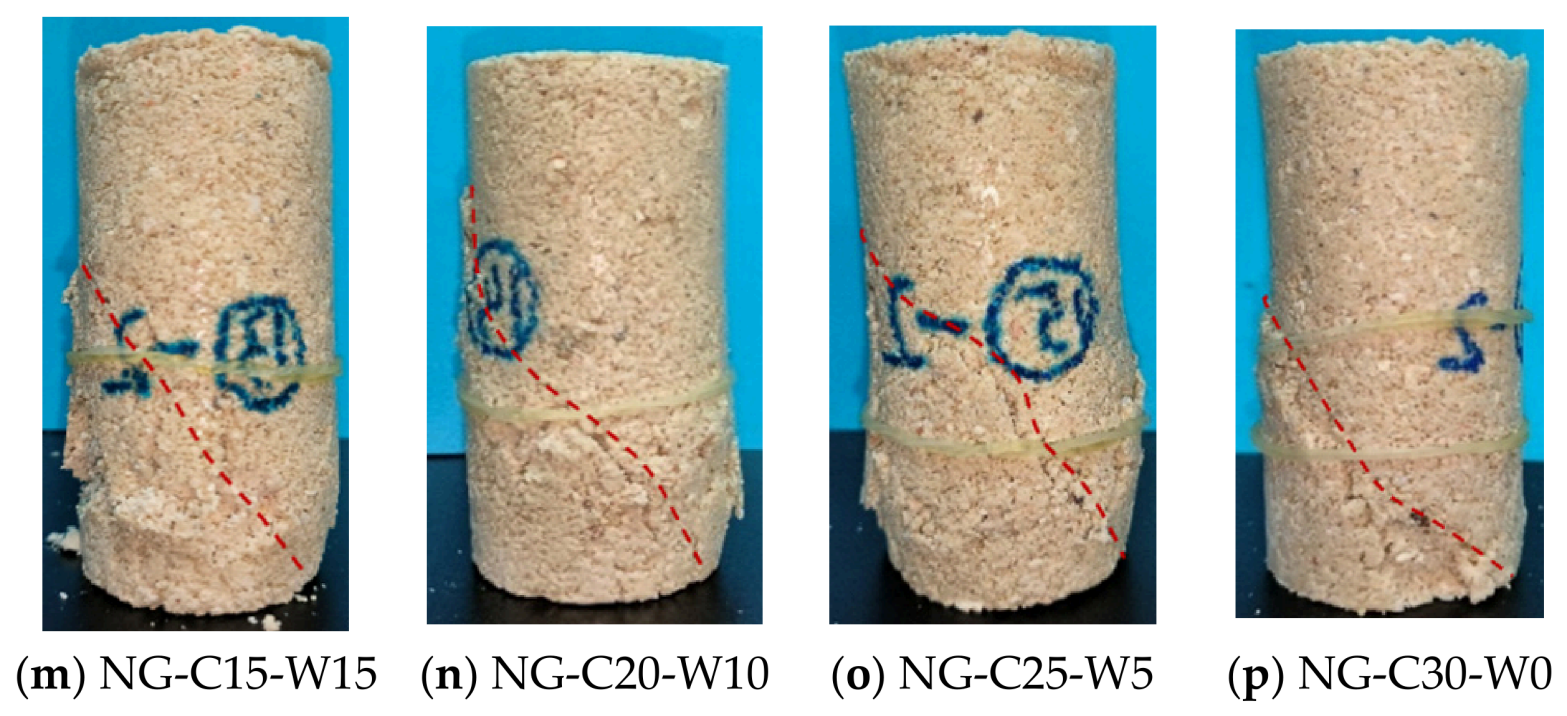
Failure pattern of polyurethane-reinforced coral sand under unconfined compressive.

Figure 15 illustrates the typical failure pattern of polyurethane-reinforced coral sand samples under unconfined compression. At the initial stage of loading, fine cracks first appear at the top and bottom ends of the samples, accompanied by slight lateral bulging. As irreversible plastic deformation accumulates, these cracks gradually extend and coalesce, forming an inclined main crack along the cylindrical surface. The failure pattern is mainly characterized by oblique shear. The measured shear failure angles are mostly distributed between 31° and 62°.

Comparison of Figure 15f,g shows that, under low-moisture conditions, cracks tend to initiate at both ends of the sample and then propagate toward the center. At higher moisture contents, the crack distribution becomes more dispersed. The corresponding stress–strain curves also show that the failure strain increases with moisture content and the post-peak branch becomes smoother. The difference between Figure 15a,p is mainly related to the polyurethane mass ratio. At low mass ratios, the polymer bridges between particles are limited in both number and thickness, and the sample surface is more prone to local spalling failure. Under loading, the polyurethane film on the sample surface may first lose stability and peel off from the substrate in thin flakes, followed by loosening of the outer structure. In the stress–strain curve, this behavior corresponds to a sharp stress drop after the peak value. As the mass ratio increases, the interparticle polymer network becomes denser and more continuous, which improves interfacial strength and toughness and reduces surface spalling. The post-peak segment of the stress–strain curve therefore shows a smoother descending trend.

### 3.6. Curing and Failure Mechanisms

**Figure 16:**
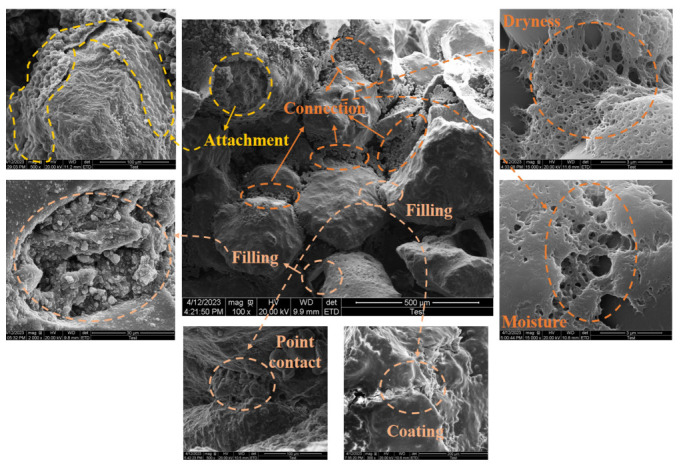
SEM of the polyurethane-reinforced coral sand.

Figure 16 presents the SEM images of polyurethane-reinforced coral sand. The SEM observations show that the cured polyurethane in the coral sand matrix presents in the forms of a thin film on particle surfaces, bridges between adjacent particles and a pore-filling phase within interparticle voids and surface pores. As shown in Figure 16, polyurethane forms a ductile three-dimensional network film after curing. The SEM observations indicate that, at lower moisture content, the polymer network is denser and more continuous, which is consistent with the previous statistical results. Coral sand particles have irregular surface morphology and abundant micropores. Together with the permeability of polyurethane, these features allow the polymer to infiltrate into the surface pores and solidify inside them. This process strengthens the interfacial adhesion between the polymer network and the sand particles and helps form a stable interface. In general, a greater amount of polyurethane material is required to achieve more complete encapsulation and pore filling.

During curing, polyurethane diffuses into and fills the voids between sand particles, forming mechanical interlocking bonds between the polymer matrix and the particle surfaces. As the mass ratio increases, the polymer gradually infiltrates and occupies the original pore network within the coral sand skeleton. At the microscopic scale, the bonding mode changes from point-contact bonding to partial surface coating and then to a continuous three-dimensional network structure. In this way, the dispersed coral sand particles are integrated into a denser composite. The reduction in interparticle pore volume also helps improve the internal stress distribution and contributes to the macroscopic mechanical properties of the material.

In summary, through the combined actions of interparticle polymer bridging, pore filling, local interfacial adhesion and mechanical interlocking, the polyurethane matrix consolidates the loose coral sand particles into a dense and continuous structure. As the mass ratio increases, both the encapsulation degree and the continuity of the polymer network are substantially improved, thereby enhancing the energy absorption capacity, deformation resistance and overall mechanical integrity of the reinforced composite. Under seawater exposure or wet–dry alternation, particle–polymer contact regions and discontinuous interparticle bridges may become preferential degradation sites. Therefore, a higher mass ratio may help reduce local debonding and maintain the integrity of the load-transfer skeleton by improving encapsulation and bridge continuity [49].

**Figure 17:**
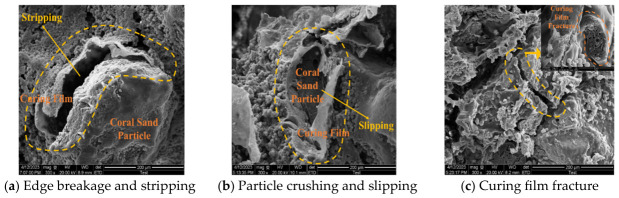
SEM of polyurethane-reinforced coral sand after failure.

The failure mechanism of the polyurethane-reinforced coral sand composite is illustrated in Figure 17. Once the sample is subjected to loading, the applied stress is first transmitted through the polyurethane spatial network structure to the coral sand particles. At low stress levels, the coral sand particles primarily undergo elastic deformation, while the binding effect of the curing film provides a confining pressure, thereby increasing the elastic limit of the particles. As the load continues to increase, the sharp edges of the coral sand particles begin to fracture, causing some particles to detach from or slip along the polyurethane film. After particle crushing occurs, the polyurethane network structure alone bears the applied load until it fractures, ultimately leading to the failure of the entire sample.

## 4. Discussion

The polyurethane-reinforced coral sand sample is a multiphase composite composed of coral sand particles, polyurethane and pores. The incorporation of polyurethane effectively fills the interparticle voids. The filling degree (*S*) is used as a quantitative indicator to characterize the effectiveness of polyurethane in filling the internal voids of coral sand [44]. It is defined as the ratio of the volume of polyurethane to the void volume of the coral sand sample, as expressed in Equation (7):

(7) $$S=\frac{V_{H}}{V_{v}}=\frac{m_{H}\rho_{s}}{em_{s}\rho_{H}}=\frac{{C\rho }_{s}}{e\rho_{H}}$$

where $V_{H}$ is the volume of polyurethane; $V_{v}$ is the pore volume between coral sand particles; *m*_H_ and *ρ*_H_ denote the mass and density of polyurethane, respectively; *e*, *m*_s_ and *ρ*_s_ represent the void ratio, mass and density of the coral sand particles; and C represents the polyurethane mixing ratio.

It is known from Equation (7) that the filling degree of the samples is primarily determined by the void ratio and mass ratio. The void ratio is influenced by gradation and relative density. Therefore, the filling degree can be used as an integrated indicator reflecting the combined effects of particle gradation and the mass ratio of polyurethane to sand. Furthermore, as discussed in the previous sections, moisture content also plays a significant role in determining the basic mechanical properties of the reinforced coral sand. Therefore, based on the two key parameters, filling degree and moisture content, a predictive analysis of the mechanical behavior of polyurethane-reinforced coral sand was conducted. Through multiple regression analysis, predictive models were established for the unconfined peak strength and elastic modulus of the reinforced samples, respectively.

To verify the interaction effects among the influencing factors, a multiple linear regression analysis was conducted. The results showed that the *p*-values for the interaction term between filling degree and moisture content were 0.31 for peak strength and 0.61 for elastic modulus. These findings indicate that the interaction between the two factors is not statistically significant (*p* > 0.05), suggesting that filling degree and moisture content are independent factors influencing the response variables, namely the peak strength and elastic modulus. Therefore, the regression equations were developed without including interaction terms. The predictive equations, representing the fitted relationships for peak strength and elastic modulus, are presented in Equations (8) and (9), with corresponding correlation coefficients *R*^2^ of 0.951 and 0.917, respectively. These coefficients indicate that the models provide a good fit to the experimental data within the range tested.

**Figure 18:**
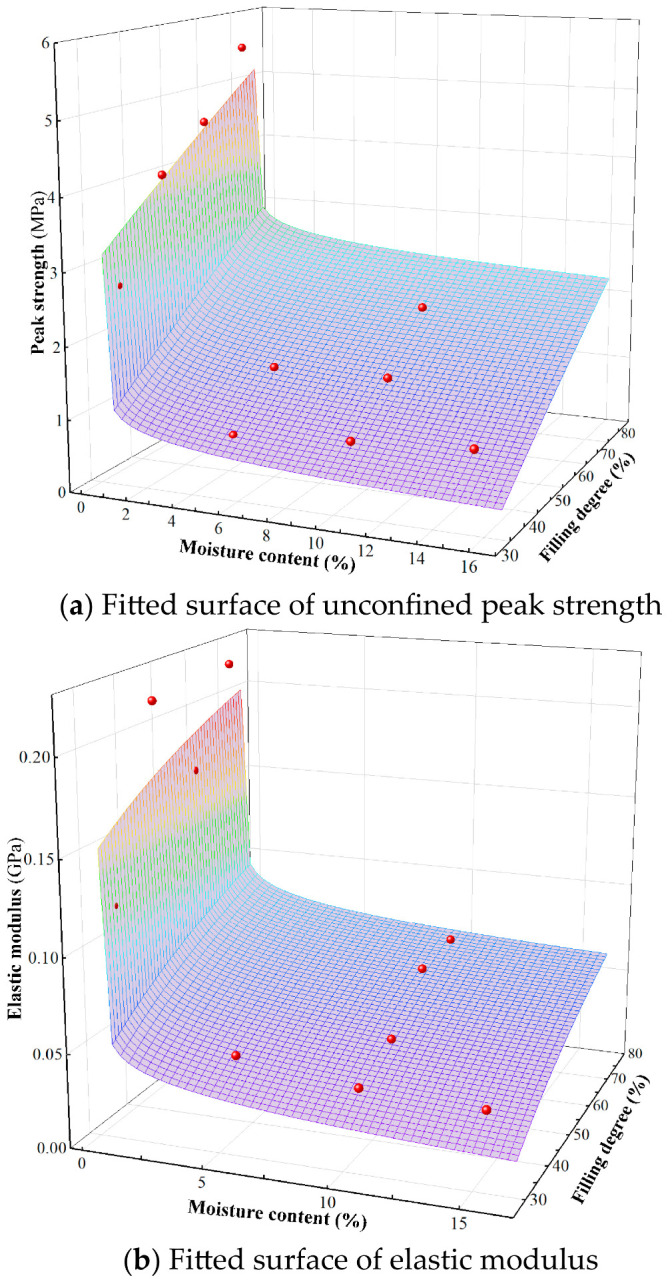
Fitted surfaces of the predictive models for the fundamental mechanical properties of polyurethane-reinforced coral sand.

Figure 18 presents the fitted surfaces corresponding to the predictive model of the fundamental mechanical properties of polyurethane-reinforced coral sand. The results indicate that the experimental data are in good agreement with the prediction equations. As shown in the figure, both the peak strength and elastic modulus of the reinforced coral sand increase significantly with the rise in filling degree. However, as the moisture content increases, the peak strength gradually decreases, with a more pronounced decline at lower moisture levels that tends to level off at higher moisture contents.

(8) $$\sigma_{p}=-1.64-0.17ln(\omega +10^{-8})+5.50S+0.75S^{2}\;(R^{2}=0.951)$$

(9) $$E_{p}=0.07-0.008ln(\omega +10^{-8})+0.06\ln S\;(R^{2}=0.917)$$

where *σ*_p_ represents the predicted peak strength, *E*_p_ denotes the predicted elastic modulus and *ω* is the moisture content.

## 5. Conclusions

Orthogonal tests of unconfined quasi-static compression on coral sand reinforced by a new polyurethane material were conducted utilizing a servo-hydraulic testing machine. The effects of gradation, moisture content and mass ratio were statistically analyzed. Furthermore, the curing and failure mechanisms of the reinforced samples were elucidated from both macro- and micro-perspectives. The conclusions are as follows:

The stress–strain response of polyurethane-reinforced coral sand can be divided into four stages: the compaction stage, elastic deformation, crack propagation and post-peak failure deformation. The highest peak strength of 5.48 MPa was obtained for the naturally graded sample with a polyurethane-to-sand mass ratio of 30% under dry conditions. It also showed high crack initiation stress, dilation strength, elastic modulus, absorbed energy and dissipated energy, indicating that the high mass ratio and the low moisture content were the most favorable combination for short-term unconfined compressive resistance.

The moisture content and the mass ratio of polyurethane to sand were the dominant factors affecting the mechanical behavior of reinforced samples. Increasing moisture content from nil to 15% dramatically reduced the average peak strength from 4.14 MPa to 1.26 MPa and promoted greater lateral deformation. These trends indicate that moisture reduces load-bearing capacity, whereas higher polyurethane content enhances polymer bridging, pore filling, stress redistribution and damage-related energy consumption. Particle gradation solely displayed a pronounced effect on the crack initiation stress level.

SEM observations demonstrated that the cured polyurethane functioned mainly as a surface film, an interparticle bridge and a pore-filling phase in the coral sand matrix. At lower moisture content, the polymer network tended to be denser and more continuous. An increase in the polyurethane mass ratio further promotes infiltration of the polymer into the original pore network within the coral sand skeleton. At the microscale, the bonding mechanism changes from point contact cementation to partial surface coating, and then to a continuous three-dimensional network.

By introducing the filling degree as a comprehensive index to quantify the pore-filling effectiveness of polyurethane, the combined influences of particle gradation and polyurethane mass ratio are effectively captured. Incorporating moisture content, multivariate linear regression models were established to predict the unconfined peak strength and elastic modulus of polyurethane-reinforced coral sand. The model results indicate that both peak strength and elastic modulus increase significantly with increasing filling degree but decrease with higher moisture content.

## Figures and Tables

**Table 1:** Basic properties of coral sand.

Sand	Density (g/cm^3^)	*e*_max_	*e*_min_	*C*_u_	*C*_c_	*d*_50_
Poorly graded sand 1	2.796	1.481	0.894	5.584	0.893	0.63
Poorly graded sand 2	2.791	1.475	0.889	8.223	0.699	1
Well-graded sand	2.788	1.565	0.988	8.000	2.000	1.67
Naturally graded sand	2.798	1.483	0.897	4.640	0.954	0.54

**Table 2:** Orthogonal test design.

	Factor	Gradation	Polyurethane Mass Ratio	Moisture Content
Level				
1		Poorly graded sand 1 (P1)	15%	0%
2		Poorly graded sand 2 (P2)	20%	5%
3		Well-graded sand (WG)	25%	10%
4		Naturally graded sand (NG)	30%	15%

**Table 3:** Scenarios for orthogonal test.

Number	Gradation Level	Polyurethane Mass Ratio Level	Moisture Content Level
P1-C15-W0	1	1	1
P1-C20-W5	1	2	2
P1-C25-W10	1	3	3
P1-C30-W15	1	4	4
P2-C15-W5	2	1	2
P2-C20-W0	2	2	1
P2-C25-W15	2	3	4
P2-C30-W10	2	4	3
WG-C15-W10	3	1	3
WG-C20-W15	3	2	4
WG-C25-W0	3	3	1
WG-C30-W5	3	4	2
NG-C15-W15	4	1	4
NG-C20-W10	4	2	3
NG-C25-W5	4	3	2
NG-C30-W0	4	4	1

**Table 4:** Mechanical parameters of polyurethane-reinforced coral sand.

Sample Number	Crack Initiation Stress (MPa)	Dilation Strength (MPa)	Peak Strength (MPa)	Elastic Modulus (GPa)	Poisson’s Ratio	Absorbed Energy (KJ/m^3^)	Dissipated Energy (KJ/m^3^)
P1-C15-W0	0.82	2.39	2.62	0.12	0.08	46.44	13.96
P1-C20-W5	0.46	1.14	1.33	0.05	0.26	22.12	2.95
P1-C25-W10	0.73	1.39	2.06	0.07	0.30	49.05	17.88
P1-C30-W15	0.97	1.50	1.92	0.06	0.26	52.5	22.16
P2-C15-W5	0.33	0.63	0.77	0.04	0.26	10.09	4.49
P2-C20-W0	2.22	3.77	3.93	0.21	0.06	56.48	20.02
P2-C25-W15	0.67	1.11	1.30	0.04	0.31	40.44	19.18
P2-C30-W10	1.29	2.01	2.17	0.07	0.29	48.71	14.49
WG-C15-W10	0.32	0.62	0.83	0.04	0.22	10.14	0.55
WG-C20-W15	0.57	0.79	0.86	0.03	0.19	22.35	9.97
WG-C25-W0	2.56	3.48	4.51	0.17	0.06	84.42	23.07
WG-C30-W5	1.18	1.99	2.20	0.07	0.14	58.31	22.88
NG-C15-W15	0.39	0.63	0.94	0.03	0.27	19.19	6.31
NG-C20-W10	0.73	1.05	1.36	0.04	0.26	27.26	6.83
NG-C25-W5	0.63	1.55	1.65	0.05	0.15	38.06	11.53
NG-C30-W0	2.77	5.14	5.48	0.22	0.06	138.06	57.30

**Table 5:** ANOVA results for crack initiation stress.

	Factor	SS	DOF	*F*-Value	*p*-Value	Significance
Crack initiation stress	Gradation	0.47	3	1.96	0.221	ns
	Mass ratio	2.42	3	10.04	0.009	**
	Moisture content	5.92	3	24.51	<0.001	***
	Error	0.48	6	—	—	—
	Total	9.30	15	—	—	—
Crack initiation stress level	Gradation	0.18	3	95.25	<0.001	***
	Mass ratio	0.02	3	8.88	0.013	*
	Moisture content	0.02	3	9.85	0.010	**
	Error	0.004	6	—	—	—
	Total	0.21	15	—	—	—

**Table 6:** ANOVA results for dilation strength.

	Factor	SS	DOF	*F*-Value	*p*-Value	Significance
Dilation strength	Gradation	0.54	3	1.73	0.260	ns
	Mass ratio	5.17	3	16.69	0.003	**
	Moisture content	18.89	3	60.97	<0.001	***
	Error	0.62	6	—	—	—
	Total	25.22	15	—	—	—

**Table 7:** ANOVA results for peak strength.

	Factor	SS	DOF	*F*-Value	*p*-Value	Significance
Peak strength	Gradation	0.33	3	0.72	0.574	ns
	Mass ratio	6.13	3	13.32	0.005	**
	Moisture content	22.00	3	47.85	<0.001	***
	Error	0.92	6	—	—	—
	Total	29.38	15	—	—	—

**Table 8:** ANOVA results for elastic modulus.

	Factor	SS	DOF	*F*-Value	*p*-Value	Significance
Elastic modulus	Gradation	0.001	3	0.37	0.779	ns
	Mass ratio	0.005	3	2.93	0.123	ns
	Moisture content	0.052	3	33.60	<0.001	***
	Error	0.003	6	—	—	—
	Total	0.060	15	—	—	—

**Table 9:** ANOVA results for Poisson’s ratio.

	Factor	SS	DOF	*F*-Value	*p*-Value	Significance
Apparent Poisson’s ratio	Gradation	0.02	3	4.18	0.065	ns
	Mass ratio	0.001	3	0.27	0.847	ns
	Moisture content	0.12	3	29.74	<0.001	***
	Error	0.01	6	—	—	—
	Total	0.13	15	—	—	—

**Table 10:** ANOVA results for energy dissipated.

	Factor	SS	DOF	*F*-Value	*p*-Value	Significance
Dissipated energy	Gradation	115.37	3	0.59	0.644	ns
	Mass ratio	1233.22	3	6.31	0.028	*
	Moisture content	913.33	3	4.67	0.052	ns
	Error	390.86	6	—	—	—
	Total	2652.78	15	—	—	—
Energy dissipation ratio	Gradation	0.02	3	0.59	0.643	ns
	Mass ratio	0.03	3	0.65	0.611	ns
	Moisture content	0.06	3	1.53	0.301	ns
	Error	0.08	6	—	—	—
	Total	0.2	15	—	—	—

## Data Availability

The data presented in the tables of this study are available within the article. The data supporting the graphs presented in this study are available from the corresponding authors upon reasonable request.
